# Supplementary material for: Anti-EGFR Therapy Induces EGF Secretion by Cancer-Associated Fibroblasts to Confer Colorectal Cancer Chemoresistance
Source: Cancers (Basel). 2020 May 28;12(6):1393. doi: 10.3390/cancers12061393 (PMC7352975; doi:10.3390/cancers12061393)
Supplement: Supplementary file 1 [file cancers-12-01393-s001.pdf]

cancers MDPI

Supplementary Materials

Anti-EGFR Therapy Induces EGF Secretion by Cancer-Associated Fibroblasts to Confer Colorectal Cancer Chemoresistance

Colleen M. Garvey [1], Roy Lau [1], Alyssa Sanchez [1], Ren X. Sun [1], Emma J. Fong [1], Michael E. Doche [1], Oscar Chen [1], Anthony Jusuf [1], Heinz-Josef Lenz [2], Brent Larson [3] and Shannon M. Mumenthaler [1,2,*]

[1] Lawrence J. Ellison Institute for Transformative Medicine, University of Southern California, Los Angeles, CA 90033, USA; colleenmgarvey@gmail.com (C.M.G.); rlau@ellison.usc.edu (R.L.); asanchez@ellison.usc.edu (A.S.); rsun@ellison.usc.edu (R.X.S.); efong@ellison.usc.edu (E.J.F.); mdoche@ellison.usc.edu (M.E.D.); oscarchen12@gmail.com (O.C.); ajusuf@usc.edu (A.J.)

[2] Division of Medical Oncology, Norris Comprehensive Cancer Center, Keck School of Medicine, University of Southern California, Los Angeles, CA 90033, USA; lenz@med.usc.edu

[3] Department of Pathology and Laboratory Medicine, Cedars-Sinai Medical Center, Los Angeles, CA 90048, USA; Brent.Larson@cshs.org

* Correspondence: smumenthaler@ellison.usc.edu; Tel.: 323-442-2529

Table S1. Clinical details of patient samples. Molecular information pertains to what was detected in the tumor epithelial cells. Cancer-associated fibroblasts are presumed to not carry oncogenic mutations.

Patient-Derived Cell Model	Sample ID#	Resection Site	Molecular Information of Patient Tumor	Clinical Treatment
Organoid	12620	liver	CK20, Villin positive; *TP53*, *PI3KCA* mutated	n/a
Normal Fibroblast (NF)	12737	colon	n/a	n/a
CAF	12905	liver	*SMARCA4*, *TSC2*, *TP53* mutated	Adjuvant FOLFOX + Avastin
CAF	12911	liver	*CHEK2* mutated	Adjuvant Xelox, switched to FOLFOX + Erbitux
CAF	13000	liver	CK7, CK20, CDX-2 positive; *KRAS* mutated	Neoadjuvant FOLFOX + Avastin

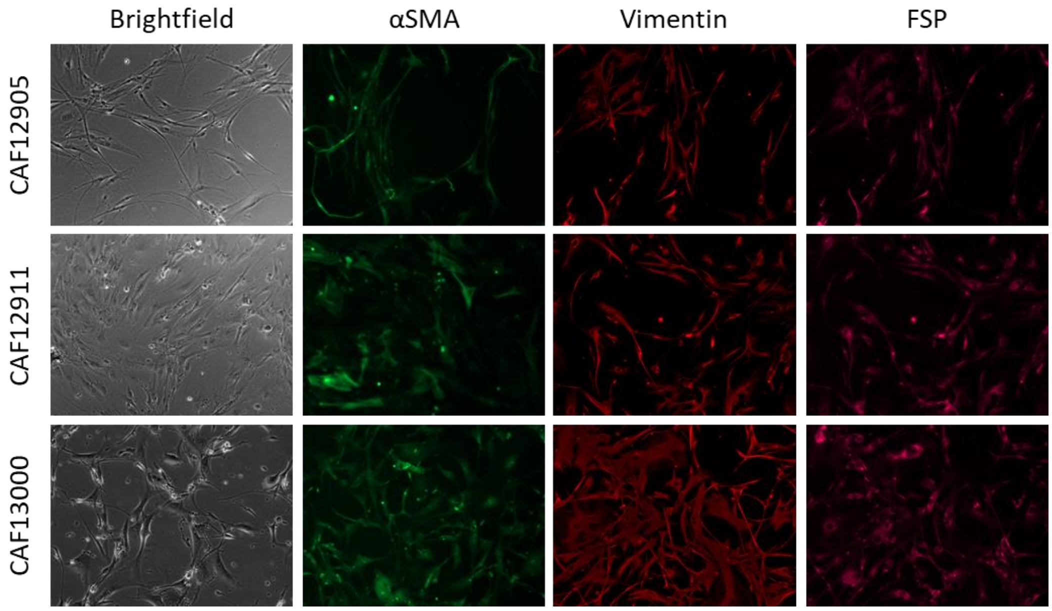

Figure S1. Validation of CAF isolation from patient tumors. Protein expression of various CAF-associated markers alpha-smooth muscle actin (αSMA), vimentin (VIM), and fibroblast specific protein (FSP) was evaluated via immunofluorescence on primary CAF lines (from patients 12905, 12911, 13000).

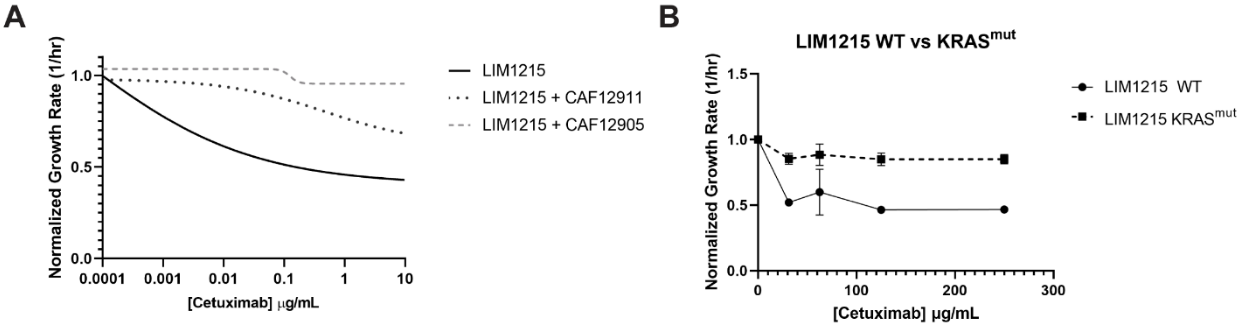

Figure S2. Cetuximab treatment of LIM1215. **(A)** LIM1215 are also de-sensitized to cetuximab treatment in the presence of CAFs. CAFs and LIM1215 cancer cells were co-cultured and treated with various concentrations of cetuximab. Starting fraction of CAFs and cancer cells was quantified on images taken prior to dosing. Growth rates of LIM1215 cells were calculated on co-cultures with CAF starting percentages ~50% by fitting live and dead cell counts taken on days 0, 3, and 5 to an exponential growth model. **(B)** Isogenic cell lines of LIM1215 KRAS wild-type (WT) and KRAS mutant ($KRAS^{mut}$) lines treated with various concentrations of cetuximab. Growth rates were normalized to untreated conditions respectively.

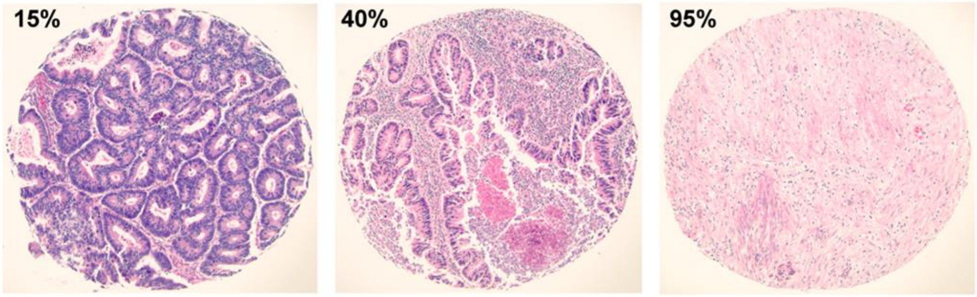

Figure S3. H&E images scored for stromal percentage. Colorectal tumor tissues were resected from treatment naïve KRAS WT metastatic CRC patients and H&E staining was performed. A pathologist estimated stromal percentage and representative images of low, medium, and high percentages are shown, with stromal percentage score annotated in the upper left corner.

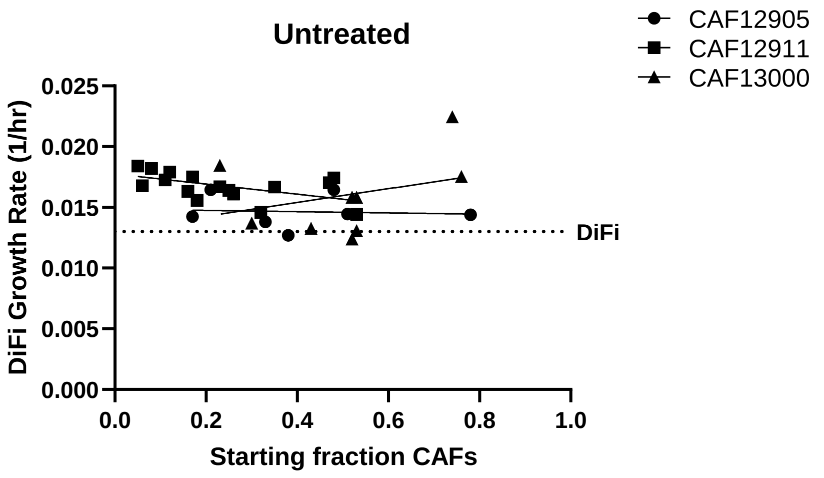

Figure S4. CAF percentage does not correlate with increased growth rate in untreated conditions. Starting ratios of CAF and DiFi cells was calculated over the course of 5-days and DiFi cell growth rates were calculated. The dotted line represents the growth rate of DiFi monoculture. R^2 values for linear fit: CAF 12905= 0.005; CAF 12911= 0.301; CAF 13000= 0.115.

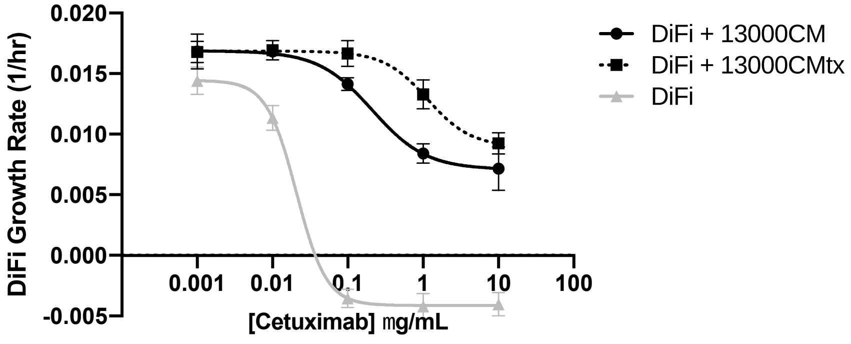

Figure S5. Dose-response curve showing conditioned media from cetuximab-treated CAFs (CMtx) is more protective than conditioned media from untreated CAFs (CM). Conditioned media was collected from primary CAFs untreated (13000CM) and treated with 1 μg/mL cetuximab (13000CMtx) for 3 days. DiFi cells were then cultured with the conditioned media conditions with or without cetuximab treatment for 5 days and growth rates were calculated.

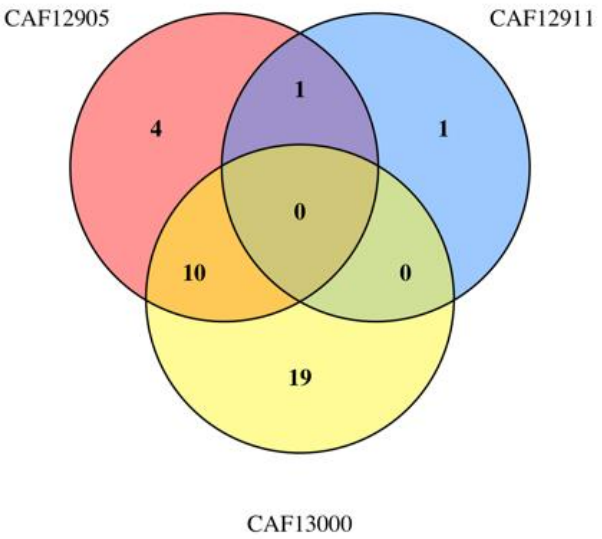

Figure S6. Comparison of downregulated cytokines from array performed on untreated and cetuximab treated CAFs. Conditioned media was collected from CAFs treated with 1 μg/mL cetuximab or IgG control for 72 hours. Cytokine expression was evaluated via cytokine arrays. Downregulated cytokines (> 0.5 fold) were determined and displayed in a Venn diagram. Upregulated cytokines are shown in Figures 3A and 3B.

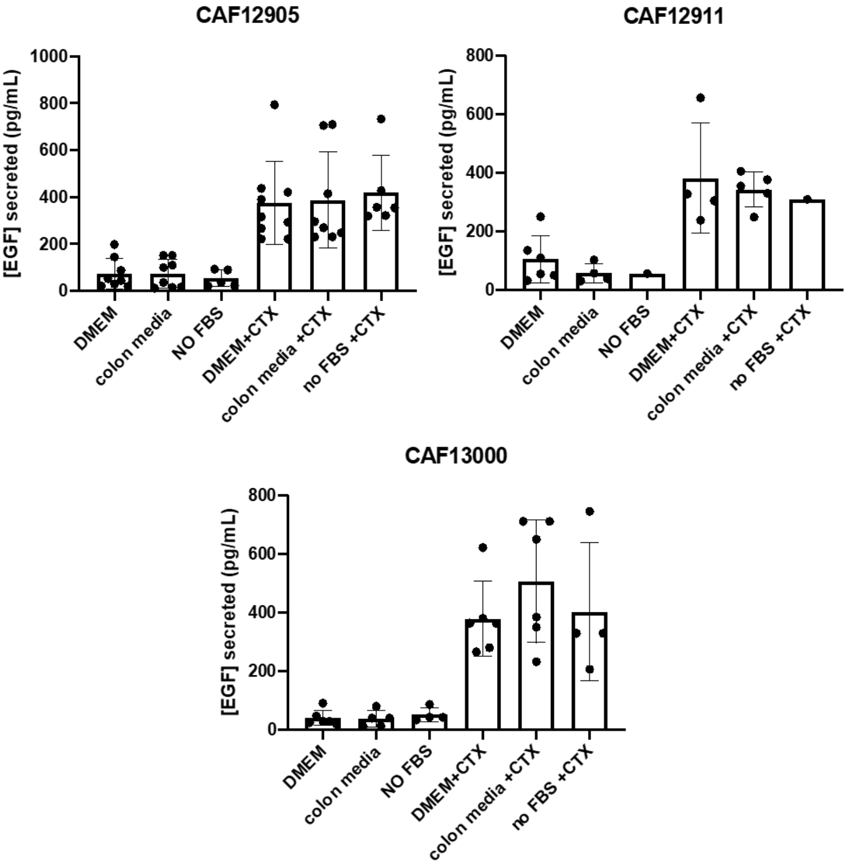

Figure S7. Media types do not change levels or patterns of EGF secretion. Primary CAFs were cultured in DMEM, defined media for culturing patient-derived colon organoids (colon media), and

FBS-free DMEM media with 1 μg/mL cetuximab or IgG control. After 72 hours, conditioned media was collected and ELISAs were performed to quantify EGF levels.

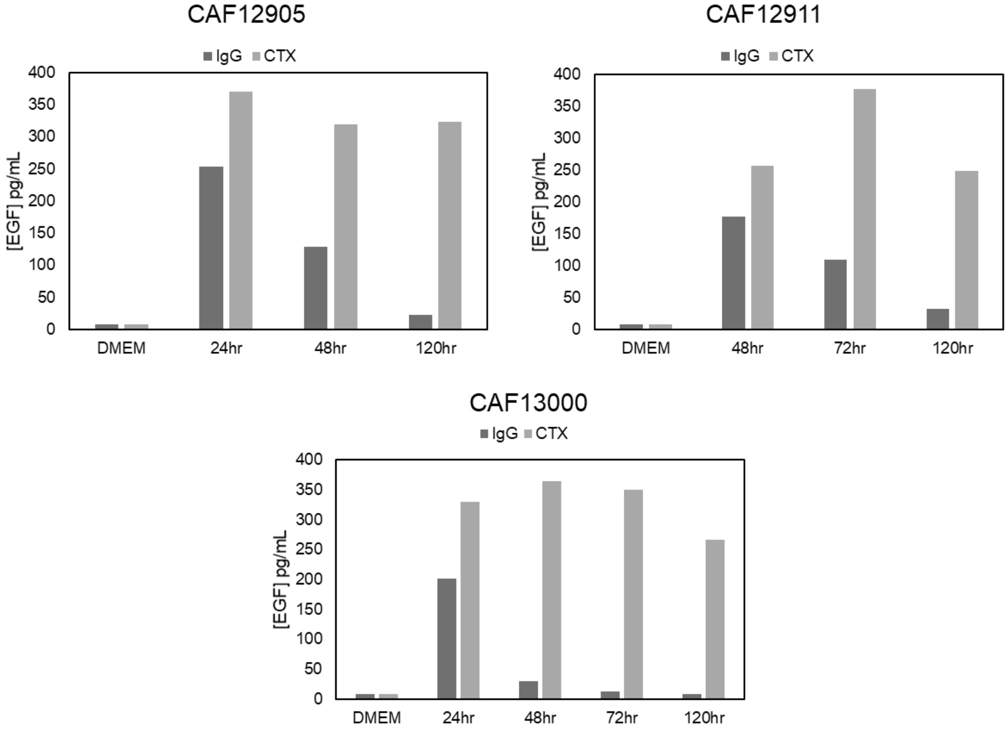

Figure S8. EGF secretion by cetuximab treated CAFs is maintained over five days. Conditioned media was collected from CAFs treated with 1 μg/mL cetuximab or IgG control for the stated time points and underwent ELISA analysis for EGF levels. The concentration of EGF in DMEM not conditioned by CAFs was also evaluated.

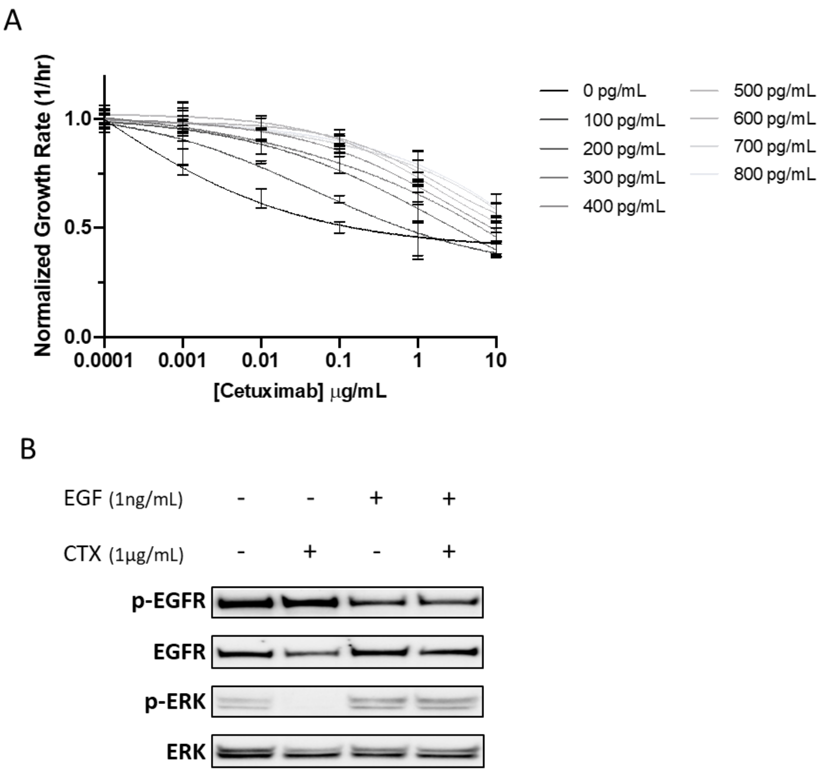

Figure S9. Exogenous EGF protects LIM1215 cells from cetuximab treatment. **(A)** LIM1215 cells were treated with cetuximab in media containing various spike-in levels of EGF. Images were acquired on days 0, 3 and 5. Live and dead cell counts were obtained and fitted to an exponential growth model to calculate the growth rate. **(B)** After being serum-starved overnight, LIM1215 cells were treated with 1 μg/ml cetuximab and/or 1 ng/ml of EGF for 2 hours. Protein expression was evaluated by Western blot.

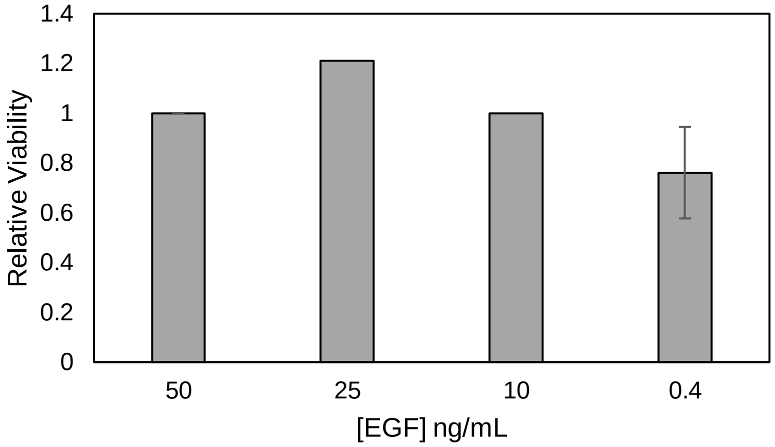

Figure S10. ORG12620 viability not altered under various EGF concentrations. Patient-derived colon tumor organoid line ORG12620 was cultured in PDO defined media containing various levels of EGF. Viability was assessed via CellTiter-Glo and normalized to 50 ng/mL condition (the normal level used for maintenance).

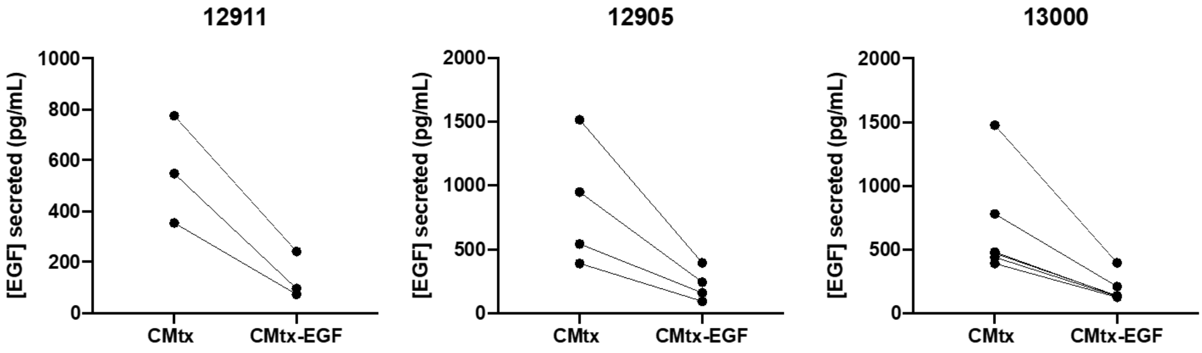

Figure S11. Validation of EGF neutralization by ELISA. ELISAs measuring EGF concentration were performed on cetuximab treated CAF conditioned media (CMtx) and CMtx treated with EGF neutralizing antibody (CMtx-EGF) used in assays found in Figure 5.

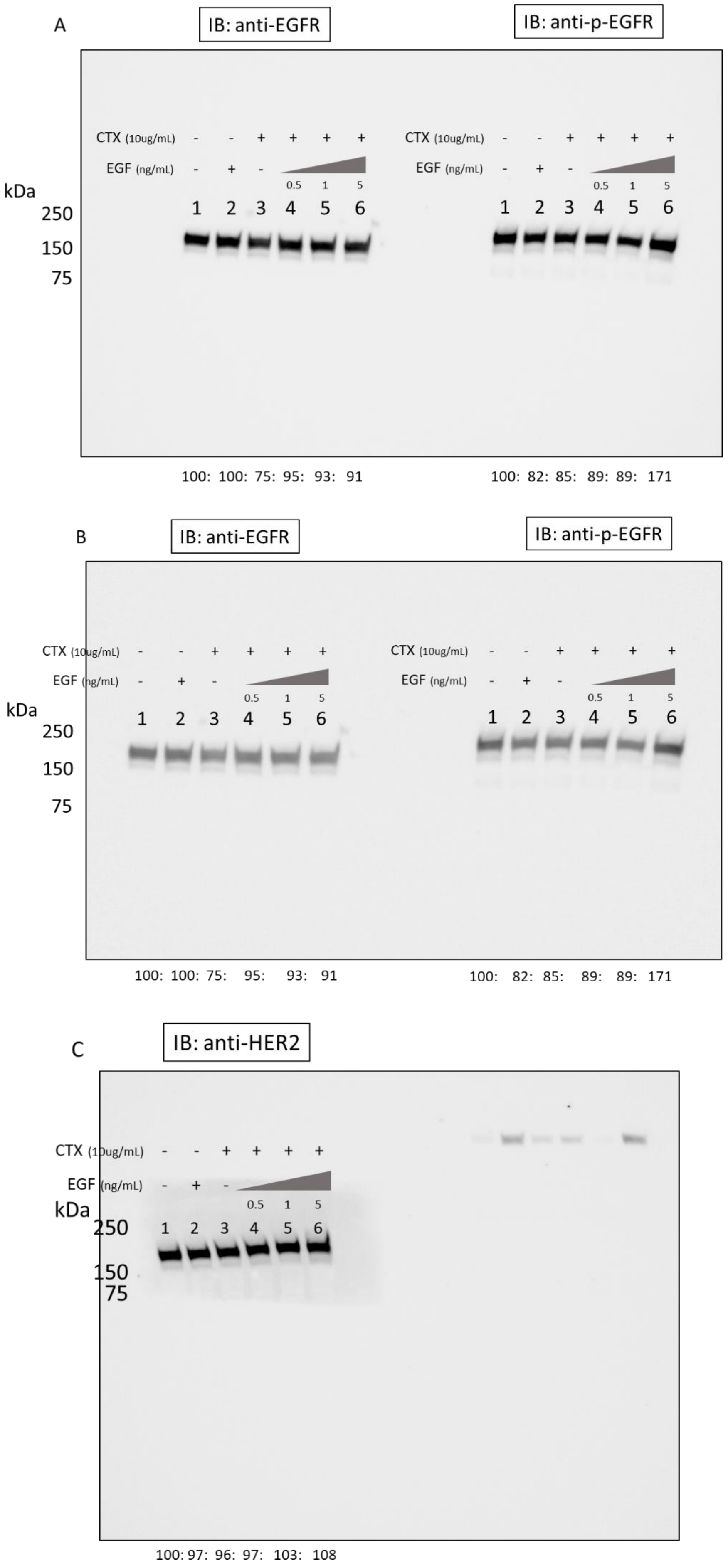
A
IB: anti-EGFR
IB: anti-p-EGFR
CTX (10ug/mL) - - + + + +
EGF (ng/mL) - + -
0.5 1 5
kDa
250
150
75
1 2 3 4 5 6
100: 100: 75: 95: 93: 91
100: 82: 85: 89: 89: 171
B
IB: anti-EGFR
IB: anti-p-EGFR
CTX (10ug/mL) - - + + + +
EGF (ng/mL) - + -
0.5 1 5
kDa
250
150
75
1 2 3 4 5 6
100: 100: 75: 95: 93: 91
100: 82: 85: 89: 89: 171
C
IB: anti-HER2
CTX (10ug/mL) - - + + + +
EGF (ng/mL) - + -
0.5 1 5
kDa
250
150
75
1 2 3 4 5 6
100: 97: 96: 97: 103: 108

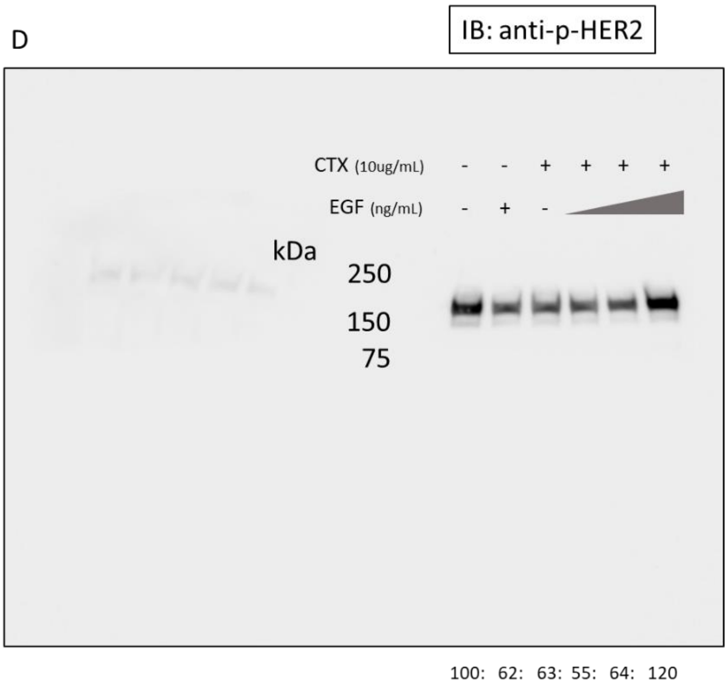
D
IB: anti-p-HER2
CTX (10ug/mL) - - + + + +
EGF (ng/mL) - + -
kDa
250
150
75
100: 62: 63: 55: 64: 120

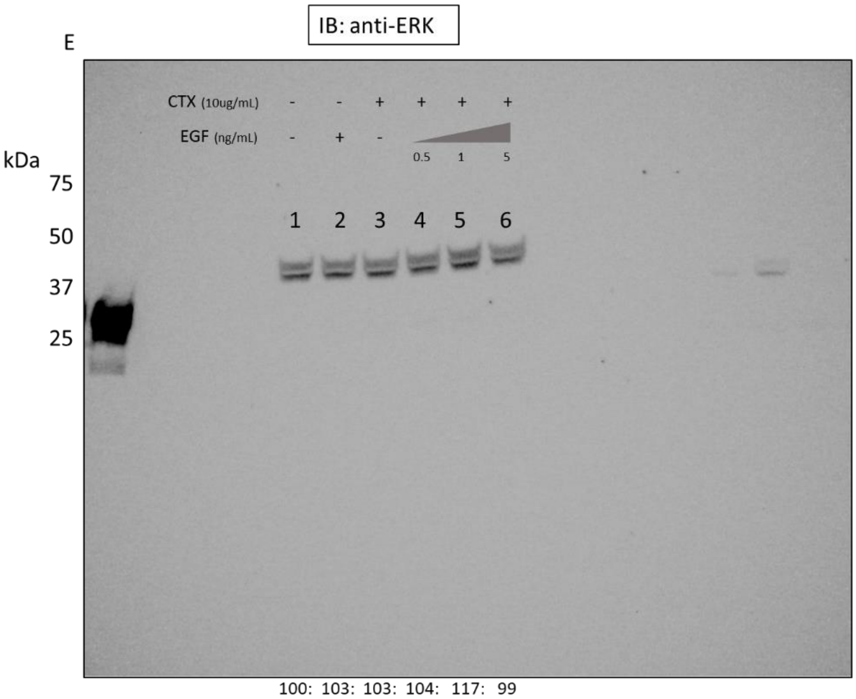
E
IB: anti-ERK
CTX (10ug/mL) - - + + + +
EGF (ng/mL) - + -
0.5 1 5
kDa
75
50
37
25
1 2 3 4 5 6
100: 103: 103: 104: 117: 99

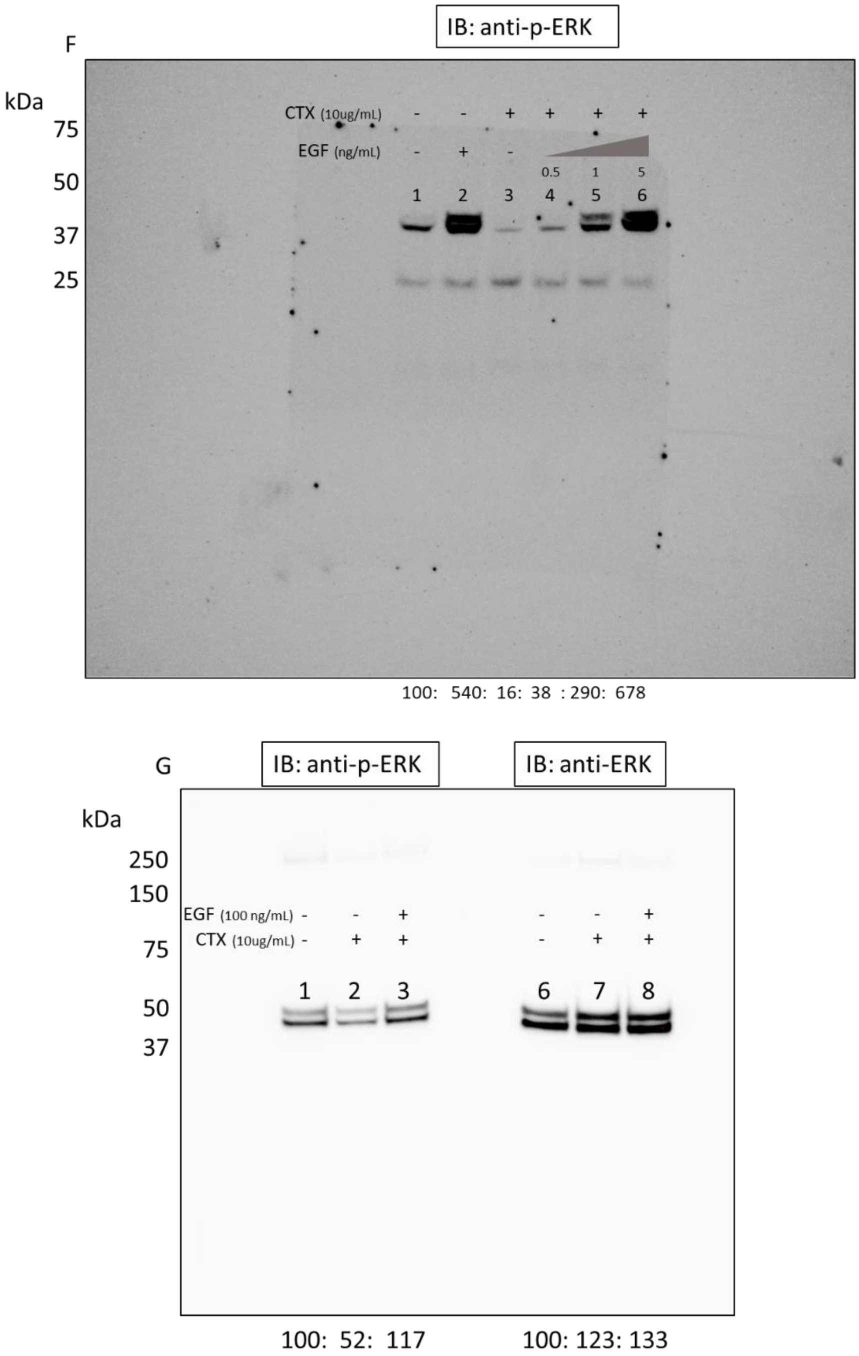
F
IB: anti-p-ERK
kDa
75
50
37
25
CTX (10ug/mL) - - + + + +
EGF (ng/mL) - + -
0.5 1 5
1 2 3 4 5 6
100: 540: 16: 38 : 290: 678
G
IB: anti-p-ERK
IB: anti-ERK
kDa
250
150
75
50
37
EGF (100 ng/mL) - - + - - +
CTX (10ug/mL) - + + - + +
1 2 3 6 7 8
100: 52: 117
100: 123: 133

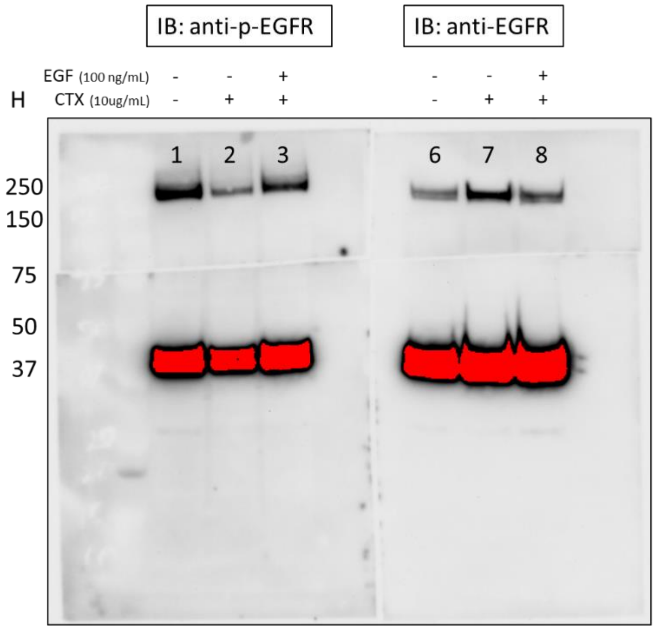
IB: anti-p-EGFR
IB: anti-EGFR
EGF (100 ng/mL) - - + - - +
H
CTX (10ug/mL) - + + - + +
1 2 3 6 7 8
250
150
75
50
37

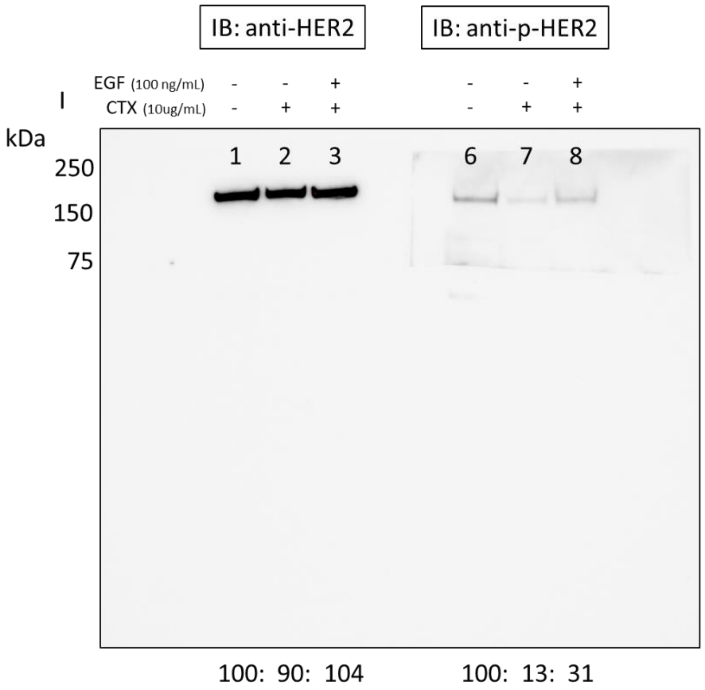
IB: anti-HER2
IB: anti-p-HER2
EGF (100 ng/mL) - - + - - +
I
CTX (10ug/mL) - + + - + +
kDa
250
150
75
1 2 3 6 7 8
100: 90: 104
100: 13: 31

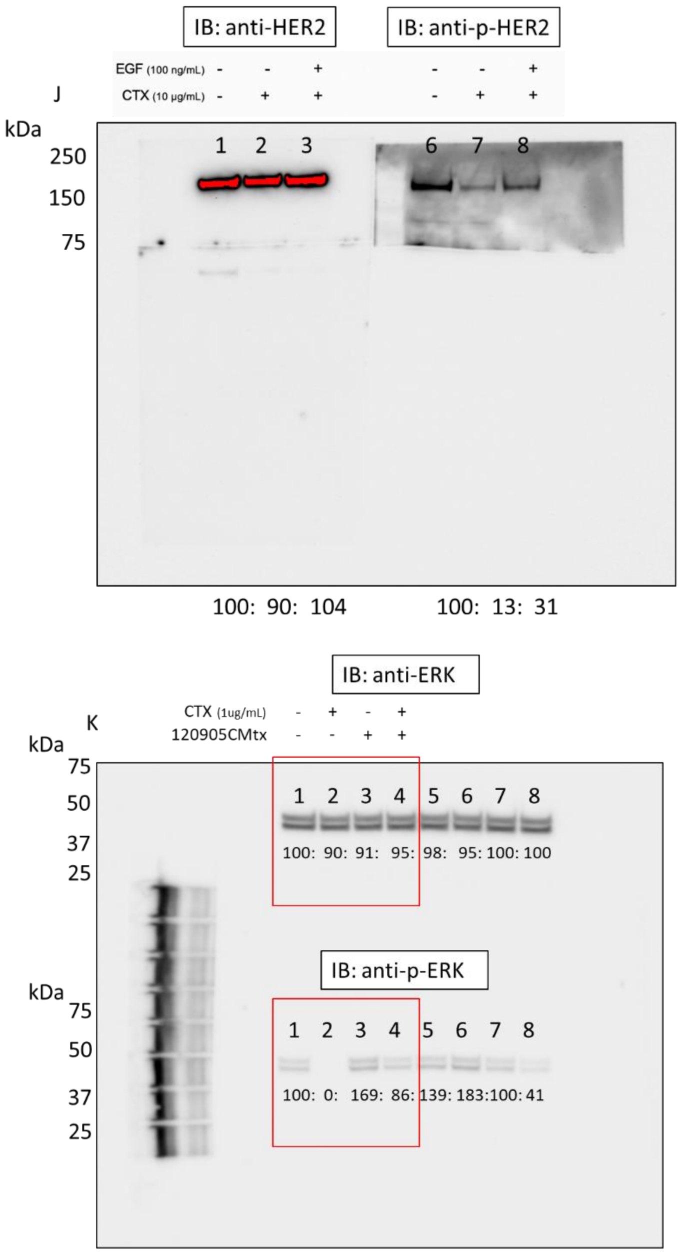

J
IB: anti-HER2
IB: anti-p-HER2
EGF (100 ng/mL) - - + - - +
CTX (10 µg/mL) - + + - + +
kDa
250
150
75
1 2 3
6 7 8
100: 90: 104
100: 13: 31
K
IB: anti-ERK
CTX (1ug/mL) - + - +
120905CMtx - - + +
kDa
75
50
37
25
1 2 3 4 5 6 7 8
100: 90: 91: 95: 98: 95: 100: 100
IB: anti-p-ERK
kDa
75
50
37
25
1 2 3 4 5 6 7 8
100: 0: 169: 86: 139: 183: 100: 41

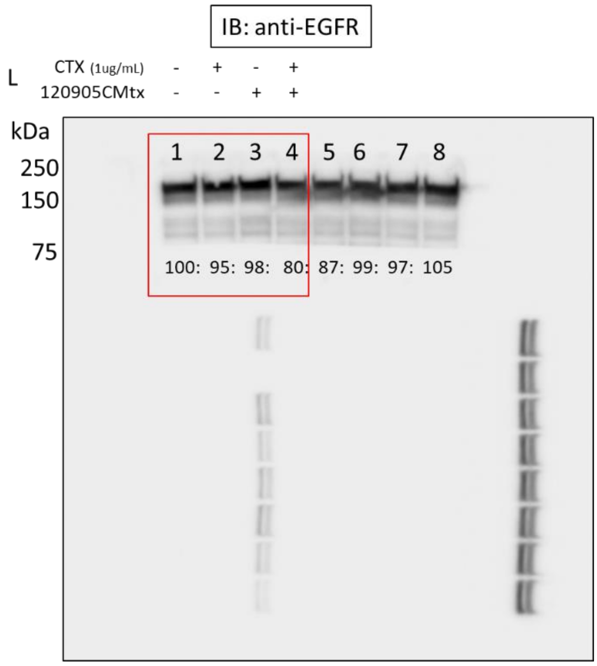
IB: anti-EGFR
L
CTX (1ug/mL) - + - +
120905CMtx - - + +
kDa
250
150
75
1 2 3 4 5 6 7 8
100: 95: 98: 80: 87: 99: 97: 105

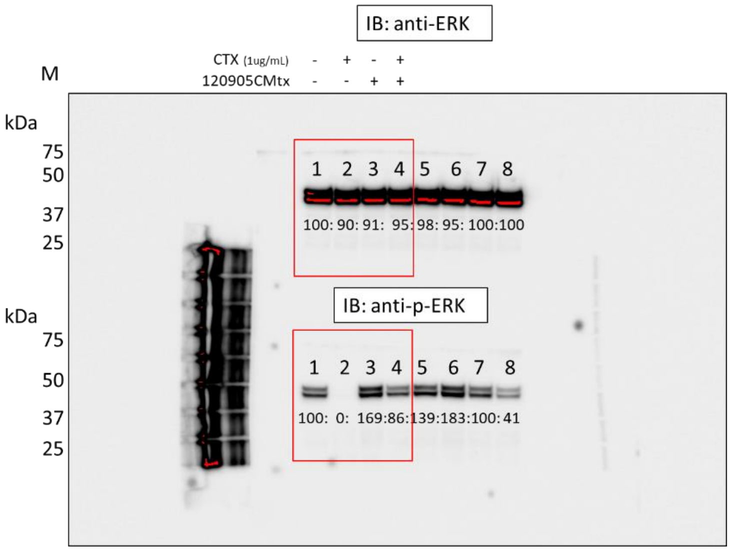
IB: anti-ERK
M
CTX (1ug/mL) - + - +
120905CMtx - - + +
kDa
75
50
37
25
1 2 3 4 5 6 7 8
100: 90: 91: 95: 98: 95: 100:100
IB: anti-p-ERK
kDa
75
50
37
25
1 2 3 4 5 6 7 8
100: 0: 169:86:139:183:100: 41

N

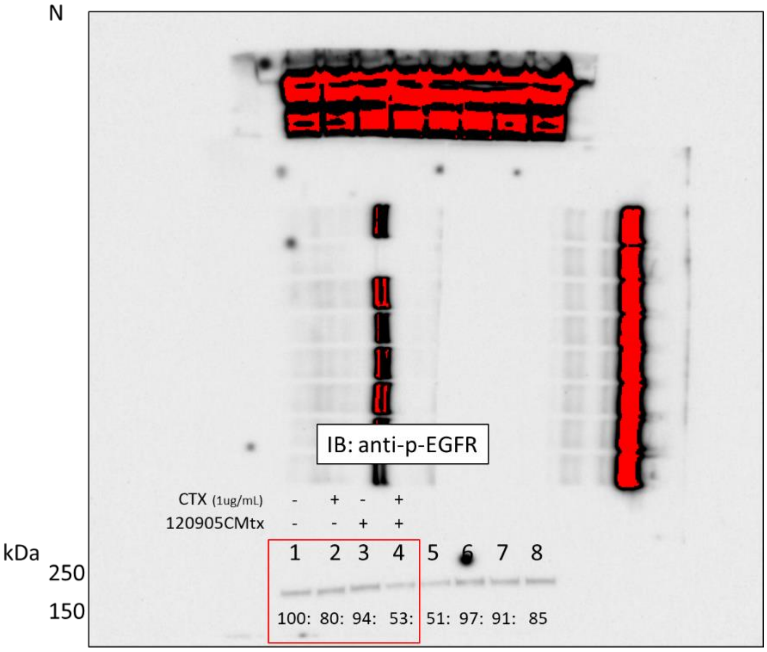
IB: anti-p-EGFR
CTX (1ug/mL) - + - +
120905CMtx - - + +
kDa
250
150
1 2 3 4 5 6 7 8
100: 80: 94: 53: 51: 97: 91: 85

O

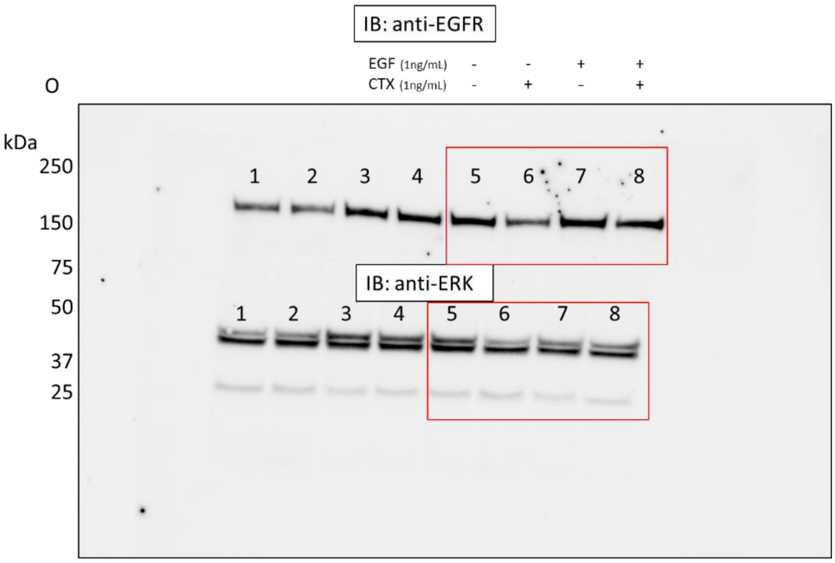
IB: anti-EGFR
EGF (1ng/mL) - - + +
CTX (1ng/mL) - + - +
kDa
250
150
75
50
37
25
1 2 3 4 5 6 7 8
IB: anti-ERK
1 2 3 4 5 6 7 8

P

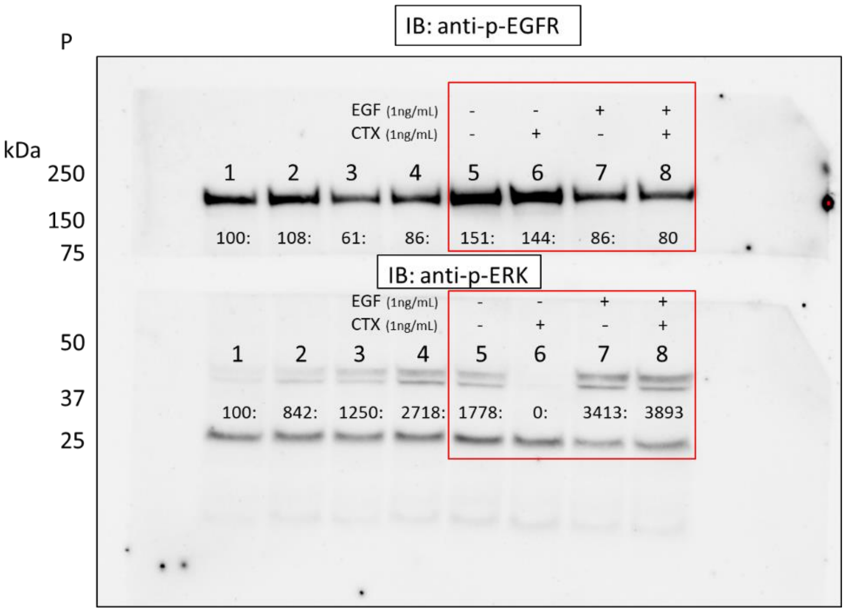
IB: anti-p-EGFR
EGF (1ng/mL) - - + +
CTX (1ng/mL) - + - +
kDa
250
150
75
1 2 3 4 5 6 7 8
100: 108: 61: 86: 151: 144: 86: 80
IB: anti-p-ERK
EGF (1ng/mL) - - + +
CTX (1ng/mL) - + - +
50
37
25
1 2 3 4 5 6 7 8
100: 842: 1250: 2718: 1778: 0: 3413: 3893

Figure S12. Uncropped originals of the Western blots depicted in the manuscript. Densitometric analysis was performed with ImageJ. **(A-F)** After being serum-starved overnight, DiFi cells were treated with 10 μg/mL cetuximab and/or increasing concentrations of EGF for 2 h. Protein expression for (A, B) EGFR and p-EGFR, (C) HER2, (D) p-HER2, (E) ERK, and (F) p-ERK was evaluated by Western blot (Reference Figure 4B in main manuscript). Band densities normalized to (-) CTX (-) EGF samples (Lane 1). **(G-J)** ORG12620 were serum-starved overnight and treated with cetuximab (10 μg/mL) and/or EGF (100 ng/mL) for 2 h. Protein expression for (G) ERK and p-ERK, (H) EGFR and p-EGFR, and (I, J) HER2 and p-HER2 was evaluated via Western blot (Reference Figure 4E in main manuscript). Band densities normalized to (-) CTX (-) EGF samples (Lane 1 for p-ERK, p-EGFR, HER2; Lane 6 for ERK, EGFR, p-HER2). **(K-N)** Conditioned media was collected from CAF12905 treated with 1 μg/mL cetuximab (12905CMtx) in fetal bovine serum (FBS)-free media. Following overnight serum-starving, DiFi cells were cultured with 1 μg/mL cetuximab and/or 12905CMtx for 2 h. Protein expression for (K) ERK, (L) EGFR, (M) p-ERK, and (N) p-EGFR was evaluated via Western blot (Reference Figure 5D in main manuscript). Note: lanes in red boxes were included in the manuscript. Other samples were for other studies. Band densities normalized to (-) CTX (-) 120905CMtx samples (Lane 1). **(O-P)** After being serum-starved overnight, LIM1215 cells were treated with 1 μg/ml cetuximab and/or 1 ng/ml of EGF for 2 hours. Protein expression for (O) EGFR and ERK, (P) p-EGFR and p-ERK was evaluated by Western blot (Reference Figure S9B in supplement). Note: lanes in red boxes were included in the manuscript. Other samples were for other studies. Band densities normalized to (-) CTX (-) EGF samples (Lane 5).
